# Comparison of pathogen detection consistency between metagenomic next-generation sequencing and blood culture in patients with suspected bloodstream infection

**DOI:** 10.1038/s41598-023-36681-5

**Published:** 2023-06-10

**Authors:** Yuhua Zhou, Wen Shi, Yi Wen, Enqiang Mao, Tongtian Ni

**Affiliations:** Department of Emergency, Ruijin Hospital, Shanghai Jiao Tong University School of Medicine, No. 197, Ruijiner Road, Huangpu District, Shanghai, 200025 China

**Keywords:** Microbiology, Health care, Medical research

## Abstract

The application of metagenomic next-generation sequencing (mNGS) has gradually been carried out by clinical practitioner. However, few studies have compared it with blood cultures in patients suffering from suspected bloodstream infections. The purpose of this study was to compare the detection of pathogenic microorganisms by these two assays in patients with suspected bloodstream infection. We retrospectively studied patients with fever, chills, antibiotic use for more than 3 days, suspected bloodstream infection, and admission to the emergency department of Ruijin Hospital from January 2020 to June 2022. All patients had blood drawn on the same day for blood mNGS and blood cultures. Clinical and laboratory parameters were collected on the day blood was drawn. The detection of pathogenic microorganisms by the two methods was compared. Risk factors and in-hospital mortality in patients with bloodstream infections were analysed separately for these two assays. In all 99 patients, the pathogenic microorganisms detection rate in blood mNGS was significantly higher than that in blood culture. Blood mNGS was consistent with blood culture in only 12.00% of all positive bacterial and fungal test results. The level of CRP is related to bacteraemia, fungaemia and viraemia detected by blood mNGS. No clear risk factors could be found in patients with a positive blood culture. In critically ill patients, both tests failed to improve patient outcomes. In patients with suspected bloodstream infection, mNGS is not yet a complete replacement for blood cultures.

## Introduction

Bloodstream infection (BSI) is an infection caused by pathogens invading the bloodstream and spreading with blood, and BSI can manifest as bacteraemia, fungaemia, and viraemia. It is a systemic infectious disease that can develop into sepsis in severe cases, causing shock, disseminated intravascular coagulation and multiple organ failure. The incidence of BSI ranges from 113 to 204 per 100,000 population^1,2^ and has been increasing yearly^3,4^. Bloodstream infection has a high fatality rate, prolongs hospitalization time, increases hospitalization costs, and causes serious harm^2,5,6^. Therefore, in addition to prevention, early identification of pathogens has received increasing attention.

Compared with traditional blood culture, metagenomic next-generation sequencing (mNGS) has the advantages of a wide range, high speed and no need for culture in diagnosing pathogenic microorganisms through high-throughput sequencing^7^. However, its use in bloodstream infections has not been popularized due to its high price. Few studies have explored the results of mNGS and blood culture assays. The aim of this study was to compare the detection of pathogenic microorganisms by these two assays in patients with suspected bloodstream infection.

## Methods

### Study subjects

We retrospectively studied patients with suspected bloodstream infection admitted to the emergency department of Ruijin Hospital from January 2020 to June 2022.

The inclusion criteria were as follows: all patients were ≥ 16 years old and meanwhile, had a maximum body temperature higher than 38.5 °C, chills, and antibiotic use for more than 3 days. Blood mNGS testing was performed on the day of blood culture collection. This study was approved by the Ruijin Hospital Ethics Committee affiliated to Shanghai Jiao Tong University School of Medicine and granted waiver of informed consent. Data analysis was performed in accordance with the ethical standards laid down in the “Declaration of Helsinki 1964” and its later amendments or comparable ethical standards.

### Blood cultures

A set of aerobic and anaerobic bottle blood samples were collected from two different parts of the patient using aseptic methods and cultured for 5–7 days, when the body temperature was ≥ 38.5 °C. The blood collection volume of each bottle was about 5–10 ml. Blood cultures were performed according to standard operating procedures of clinical microbiology. The fully automated blood culture apparatus used was BACTECFX (Becton, Dickinson and Company, US). Bacterial identification was performed by using the VITEK MS detection system (BioMérieux, Marcyl’Étoile, France). A positive blood culture was defined as the detection of a specific pathogen such as bacteria or fungi in the blood, while a negative blood culture was defined as the absence of any organisms growing during the incubation period. Whether blood cultures were considered contaminated was defined by two associate chief physicians.

### Metagenomic next-generation sequencing

Approximately 6–8 mL of blood was collected from each patient. The samples were cold preserved on dry ice and transferred to Shanghai Hugo Biotech Co., Ltd. or Guangzhou Vision Medicals Co., Ltd. for testing. Test results were provided the day after the samples arrived.

### Testing process of Shanghai Hugo Biotech Co., Ltd.

We used techniques reported in the previous literature^8^. The QIAamp DNA Micro Kit (QIAGEN, Germany) was used to extract DNA from a sample, and the extracted DNA was used to construct DNA libraries using the QIAseq™ Ultralow Input Library Kit for Illumina (QIAGEN, Germany). The quality of the libraries was assessed using a Qubit (Thermo Fisher) and Agilent 2100 Bioanalyzer (Agilent Technologies), and the qualified libraries were then sequenced on the Nextseq 550 platform (Illumina). To obtain high-quality data, adapter, short, low-quality, and low-complexity reads were removed from the raw data, and human host DNA reads were filtered out by alignment to the human reference database (hg38). The remaining reads were screened or aligned to the Microbial Genome Databases (ftp://ftp.ncbi.nlm.nih.gov/genomes/) using the Burrows-Wheeler Aligner software^9^. To report a positive mNGS result for detected microbe reads, including bacteria (excluding *Mycobacteria*), fungi (excluding *Cryptococcus*), and parasites, the coverage of the microbe had to rank in the top 10 of the same kind of microbe and be absent in the negative control ("No template" control, NTC), or the ratio of reads per million (RPM) between the sample and the NTC (RPMsample/RPMNTC) had to be > 10 if RPMNTC was not equal to 0. For viruses, *M. tuberculosis*, and *Cryptococcus*, a positive mNGS result was considered when at least 1 unique read was mapped to the species level and absent in the NTC or when RPMsample/RPMNTC > 5 when RPMNTC was not equal to 0^8^.

### Testing process of Guangzhou Vision Medicals Co., Ltd.

We used techniques reported in the previous literature^10^. The DNA was extracted using a QIAamp® Microbiome DNA Kit, and a Nextera XT DNA Library Prep Kit^11^ was used to construct a library of the DNA. Quality control was performed using a Qubit dsDNA HS Assay Kit and High Sensitivity DNA kit (Agilent) on an Agilent 2100 Bioanalyzer. The library pools were then sequenced using an Illumina NextSeq CN500 sequencer, and the resulting data were processed using Trimmomatic^12^ to remove low quality reads, adapter contamination, duplicated reads, and reads shorter than 50 bp. To identify human sequences, the remaining data were mapped to a human reference (hg19) using Burrows-Wheeler Aligner software^9^ and were excluded. The remaining sequence data were then aligned to bacterial, virus, fungal, and protozoan databases (NCBI; ftp://ftp.ncbi.nlm.nih.gov/genomes). Unique reads were defined as reads whose alignment length was higher than 80%, identity with reference sequence was higher than 90%, and ratio of suboptimal to optimal alignment score was lower than 0.8. A positive detection was reported for a given species or genus if the RPM ratio, or RPM-r was ≥ 10, where the RPM-r was defined as the RPMsample / RPMNTC (the RPM corresponding to a given species or genus in the clinical sample divided by the RPM in the NTC). A whole blood sample from healthy donors was prepared alongside each batch using the same extraction protocol as a negative control to account for any potential contamination^10^.

### Clinical variables

Electronic medical records of all enrolled patients were reviewed and data on the day of testing were obtained, including demographic information, laboratory tests (white blood cell, haemoglobin, platelet, C-reactive protein, procalcitonin, alanine aminotransferase, albumin, total bilirubin, blood urea nitrogen, serum creatinine, and lactic acid), comorbidities (hypertension, diabetes mellitus, valvular heart disease, heart failure, atrial fibrillation, chronic pulmonary disease, chronic liver disease, chronic kidney disease, coagulopathy, rheumatic disease, and tumor), clinical evaluation (central venous catheter, mechanical ventilation, Sequential Organ Failure Assessment (SOFA) score, and in-hospital mortality).

### Statistical analysis

The clinical data were performed by using SPSS 26.0 statistical software (SPSS, Inc., Chicago, IL). The chi-square test was used to compare categorical variables. Binary logistic regression analysis was performed of risk factors for positive blood mNGS and blood culture in patients with suspected bacteraemia. The enumeration data are presented as the means (x̅) ± standard deviation (SD) or medians (interquartile range) while categorical data are expressed as frequencies and percentages. P < 0.05 was considered to be threshold of statistical significance.

## Results

### Characteristics of the study population

A total of 99 patients with suspected bloodstream infection aged from 16 to 88 years old were formally enrolled in our study. Baseline characteristics for the patients are shown in Table 1. All enrolled patients were from the emergency department of Ruijin hospital, and their comorbidities involved various specialties. The mean or quartile of inflammatory indicators including WBC, CRP, and PCT were all higher than the normal range. The proportions of central venous intubation and mechanical ventilation were 56.57% and 36.36%, respectively. The median SOFA score was 6 points. The in-hospital mortality rate was 37.37%.

**Table 1:** Clinical characteristics on the day of blood mNGS and blood culture testing in patients with suspected bloodstream infection (n%; $\overline{x }$ ± s; median (IQR)).

	Patients with suspected bloodstream infection (n = 99)
Mean age (years)	63.00 (49.00, 72.00)
Gender (male %)	74 (74.75%)
Body mass index (kg/m^2^)	23.78 (22.04, 25.95)
Current smoker (%)	29 (29.29%)
Alcohol abuse (%)	15 (15.15%)
Indicators	
White blood cell (× 10^9^/l)	10.58 ± 6.07
Hemoglobin (g/l)	95.23 ± 23.26
Platelet (× 10^9^/l)	111.00 (43.00, 180.00)
C-reactive protein (µg/ml)	99.00 (40.00, 208.00)
Procalcitonin (ng/ml), median (IQR)	2.78 (0.44, 16.47)
Alanine aminotransferase (U/l), median (IQR)	31.00 (18.00, 88.00)
Albumin (g/l)	33.19 ± 6.02
Total bilirubin (µmol/l)	18.50 (12.40, 39.60)
Blood urea nitrogen (mmol/l)	9.30 (5.70, 15.60)
Serum creatinine (µmol/l)	87.00 (60.00, 212.00)
Lactic acid (mmol/l)	2.26 (1.45, 3.20)
Comorbidities	
Hypertension (%)	47 (47.47%)
Diabetes mellitus (%)	30 (30.30%)
Valvular heart disease (%)	16 (16.16%)
Heart failure (%)	23 (23.23%)
Atrial fibrillation (%)	7 (7.07%)
Chronic pulmonary disease (%)	13 (13.13%)
Chronic liver disease (%)	8 (8.08%)
Chronic kidney disease (%)	13 (13.13%)
Coagulopathy (%)	6 (6.06%)
Rheumatic disease (%)	12 (12.12%)
Tumor (%)	11 (11.11%)
Central venous catheter (%)	56 (56.57%)
Mechanical ventilation (%)	36 (36.36%)
Sequential organ failure assessment score	6.00 (3.00, 9.00)
In-hospital mortality (%)	37 (37.37%)

*SOFA* sequential organ failure assessment, *IQR* interquartile range.

### Detection of pathogenic microorganisms by mNGS and traditional blood culture

**Figure 1:**
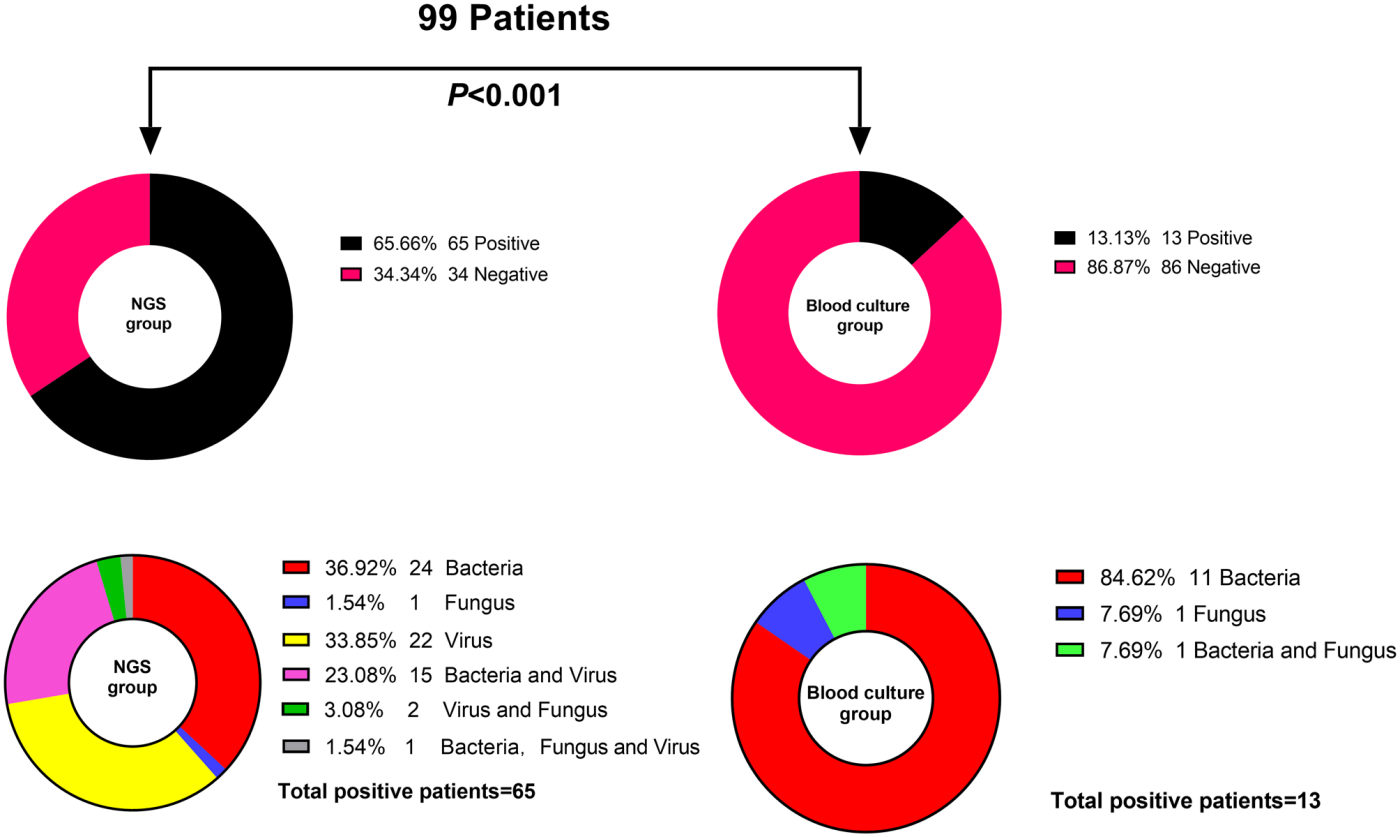
Overview of blood mNGS and blood cultures in patients with suspected bloodstream infection.

The patients’ number of mNGS-positive versus blood culture-positive was 65 versus 13 (65.66% vs. 13.13%). The difference between them showed statistical significance (P < 0.001). Among the mNGS-positive patients, 43 had bacteria and/or fungi detected, and 22 had a virus alone. Pathogenic microorganisms detected from patients with positive blood cultures were all bacteria and/or fungi (Fig. 1).

The three most common bacteria and fungi in blood cultures were *Klebsiella pneumoniae*, *Enterococcus faecium* and *Staphylococcus haemolyticus*, while *Klebsiella pneumoniae*, *Escherichia coli*, *Salmonella enterica* were most common in blood mNGS (Table 2).

**Table 2:** The top three bacteria and fungi in blood mNGS and blood culture.

	Blood culture	n = 13	Blood mNGS	n = 43
1	Klebsiella pneumoniae	3 (23.08)	Klebsiella pneumoniae	9 (20.93)
2	Enterococcus faecium	3 (23.08)	Escherichia coli	7 (16.28)
3	Staphylococcus haemolyticus	2 (15.38)	Salmonella enterica	4 (9.30)
4	Other	5 (38.46)	Other	23 (53.49)

*mNGS* metagenomic next-generation sequencing.

**Figure 2:**
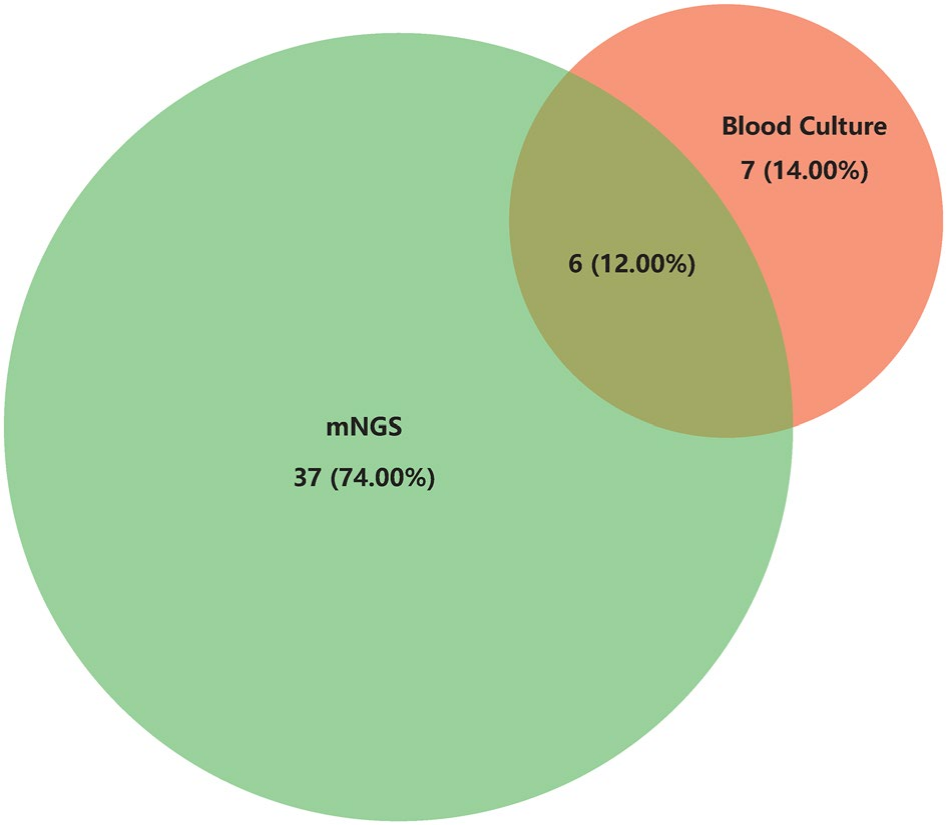
Consistency of bacteria and fungi detected in blood mNGS and blood culture.

In terms of the detection results concerning bacteria and fungi, the mNGS detection rate was significantly higher than blood culture (Table 3). A Venn diagram^13^ showed that the concordance rate between mNGS and blood culture in the detection of bacteria and fungi was 12.00% (Fig. 2).

**Table 3:** Comparison of detection rate of bacteria and fungi in blood mNGS and blood culture.

	Blood mNGS	Blood culture	*P* value
Positive	43	13	< 0.001*
Negative	56	86	

*mNGS* metagenomic next-generation sequencing.

*P < 0.05 was considered statistically significant.

### Comparison of the risk factors for positive mNGS and blood culture

By binary logistic regression, it was found that a decreased BMI, decreased WBC count and increased CRP were risk factors for the detection of pathogenic microorganisms in blood mNGS. Advanced age, elevated CRP, alcohol abuse, and rheumatic diseases are risk factors for bacterial and/or fungal detection in blood mNGS. Risk factors for positive blood cultures were gender and current smoker (Table 4).

**Table 4:** Binary logistic regression analysis of risk factors for positive blood mNGS and blood culture in patients with suspected bacteremia.

	Adjusted OR (95%CI)	*P* adjusted
mNGS (bacteremia/fungemia/viraemia)		
Body mass index	0.852 (0.747–0.973)	0.018*
White blood cell	0.872 (0.794–0.957)	0.004*
C-reactive protein	1.007 (1.001–1.013)	0.020*
mNGS (bacteremia/fungemia)		
Age	1.055 (1.022–1.190)	0.001*
C-reactive protein	1.008 (1.002–1.013)	0.008*
Alcohol abuse	5.430 (1.453–20.297)	0.012*
Rheumatic disease	7.388 (1.669–32.698)	0.008*
Blood culture (bacteremia/fungemia)		
Gender	11.901 (1.323–107.016)	0.027*
Current smoker	19.429 (2.287–165.083)	0.007*

*P* adjusted: assessed by binary logistic regression; adjusted for age, gender, body mass index, current smoker, alcohol abuse, white blood cell, hemoglobin, platelet, C-reactive protein, procalcitonin, alanine aminotransferase, albumin, total bilirubin, blood urea nitrogen, serum creatinine, lactic acid, hypertension, diabetes mellitus, valvular heart disease, heart failure, atrial fibrillation, chronic pulmonary disease, chronic liver disease, chronic kidney disease, coagulopathy, rheumatic disease, tumor, central venous catheter, mechanical ventilation, Sequential Organ Failure Assessment score.

*mNGS* metagenomic next-generation sequencing.

*P < 0.05 was considered statistically significant.

### The relationship between bloodstream infection and mortality

The in-hospital mortality rates of mNGS-positive and mNGS-negative patients were 38.46% and 35.29%, while those of blood culture-positive and blood culture-negative patients were 38.46% and 37.21%, respectively. Neither blood mNGS positivity nor blood culture positivity showed an improvement in in-hospital mortality (Table 5).

**Table 5:** The relationship between bloodstream infection and mortality.

	mNGS positive (n = 65)	mNGS negative (n = 34)	*P* value
In-hospital mortality			
n (%)	25 (38.46%)	12 (35.29%)	0.757

	Blood culture positive (n = 13)	Blood culture negative (n = 86)	*P* value
In-hospital mortality			
n (%)	5 (38.46%)	32 (37.21%)	0.931

*mNGS* metagenomic next-generation sequencing.

### Ethics approval and consent to participate

This study was approved by the Ruijin Hospital Ethics Committee affiliated to Shanghai Jiao Tong University School of Medicine and granted waiver of informed consent. Data analysis was performed in accordance with the ethical standards laid down in the “Declaration of Helsinki 1964” and its later amendments or comparable ethical standards.

## Discussion

BSI is a major global public health burden, threatening the lives of patients. Early diagnosis of bloodstream infections is very important. Blood cultures are currently still the gold standard, with results typically available in 3–5 days. Until then, broad-spectrum antibiotics are generally empirically prescribed to treat patients with suspected bloodstream infections. Limited by sensitivity and long turnaround times^14^, other detection techniques including multiplex real-time PCR working directly basing on whole blood, PCR combined with T2 Magnetic Resonance and metagenomics-based assays are rapidly developing. Among them, mNGS is the characterised by a rapid diagnosis time and can improve the management process of patients with suspected bloodstream infection^14^.

In our study, mNGS technology use often coincided with cases where the diagnosis of pathogenic microorganisms was difficult or the patient was extremely ill, and at the same time, antibiotics had been used for some time. Therefore, the duration of antibiotic treatment in the patients enrolled in this study was more than 3 days. Empirical antimicrobial therapy drawn after initial therapy significantly reduces the blood culture sensitivity^15^. Positive blood culture rates after antibiotic use ranged from 11 to 27.7%^15–18^. The positive rate of blood cultures in our study was 13.13%, which was consistent with that reported in previous studies. The low positive rate of blood cultures may also be related to the less than optimal blood collection volumes^19^. The blood cultures in our study were drawn in volumes of 5–10 ml, which was less than the recommended 10 ml or more.

Both the mNGS technique and blood culture in this study can identify bacteria and fungi, but mNGS can also identify viruses and parasites. In the analysis report, no results were found for parasites. The top three bacteria were all common bacteria, consistent with previous studies^17,20^. The sensitivity of mNGS is very high, and its negative results often have a good negative predictive value for excluding infection^21,22^. In terms of bacterial and fungal detection results, the positive rate of mNGS was approximately 3.31 (43/13) times higher than that of blood culture. The concordance of the two assays was compared in a Venn diagram and it was found that the mNGS results did not overlap the blood culture results to a large extent. In the study by Long et al.^17^, the concordance between mNGS and blood culture for bacterial and fungal detection was 8/20 (40%), which was higher than the 6/50 (12.00%) in our study. The reason for this phenomenon might be related to the machine algorithm, we only accepted pathogens with high confidence in mNGS and ignored pathogens with medium confidence. In addition, their Venn diagram only compared negatives and positives, not down to the level of pathogen species. So far, various testing methods have limitations and cannot achieve 100% sensitivity. Possible reasons for false negatives include: 1. Pathogen nucleic acid levels below the threshold of mNGS detection; 2. Sequences of the detected pathogens below the reporting threshold, which may be filtered out; 3. Pathogens not present in the bloodstream or the pathogen's nucleic acid sequences not yet entering the bloodstream; 4. Nucleic acid degradation; 5. Preemptive use of antibiotics, and so on. However, even with a 40% concordance rate, mNGS could not completely replace blood culture. To increase the positive rate of detection, mNGS needs to be used in combination with blood culture.

To our knowledge, few studies have explored risk factors for a positive blood mNGS test. Risk factors affecting mNGS bacterial and fungal test results included age, C-reactive protein, alcohol abuse, and rheumatic diseases. When viruses was added to the test results, the risk factors became body mass index, C-reactive protien and white blood cell counts. The predictive value of C-reactive protein was shown in both analyses. C-reactive protein has also been identified as one of important risk factors for positive blood cultures in sepsis patients^23,24^. Positive blood cultures were found to increase with increased procalcitonin level, degree of liver failure, and SOFA score in a retrospective study^25^. The insertion and duration of central venous catheters have also been identified as high-risk factors for BSI^26^. However, these risk factors for positive blood culture were not found in our study. This might be due to the low number of positive samples.

BSI has a high mortality rate and delaying treatment could seriously affect patient outcomes^27^. In our study, the mortality rates of mNGS and blood culture-positive patients were all 38.46%, which were higher than those of bloodstream infection patients in previous studies^3,28^. This was due to the high cost of mNGS, which is often used when conventional diagnosis and treatment was ineffective or for the identification of difficult microorganisms, so the disease situation in these selected patients was more severe. Although their median SOFA score on the day of mNGS was 6, their number of comorbidities exceeded those reported in patients with sepsis in a previous study^29^. Patients with positive blood mNGS and/or blood cultures had no significant difference in mortality compared with negative patients. This is different from previous research results indicating that the in-hospital mortality rate of positive blood culture patients was significantly increased^28^. Another study found that mNGS-positive patients had longer hospital stays and higher 28-day mortality^30^. However, these results remain controversial. The findings of Marco et al. were similar to ours, and mortality did not increase with a positive test result for bacteraemia^25^. In another prospective cohort study, positive blood cultures were not associated with outcomes in patients with sepsis^31^. A recent meta-analysis also showed that positive or negative blood cultures were not associated with mortality in patients suffering fromsepsis^32^. This might be due to the early empirical use of antibiotics in all these patients. Patients with bloodstream infections, including positive blood mNGS or positive blood cultures, are generally more severely ill. But positive patients may have a more significant remission after targeted interventions. This may account for the fact that there is no statistically significant difference in in-hospital mortality. It has been shown^33^ that early and effective antimicrobial therapy can improve patient outcomes, which may improve mortality in patients with bloodstream infections, showing no significant difference in mortality between positive and negative patients.

## Conclusion

In patients with suspected bloodstream infection, the probability of positive blood mNGS is significantly higher than that of blood culture, but it cannot completely replace the role of blood culture. The combined use of blood mNGS and blood culture could maximize the detection of pathogenic microorganisms in bloodstream infections.

### Limitations

Although the same mNGS platform technology was used, the algorithms of different companies were still different, which might lead to errors in the detection results to a certain extent. In addition, because this was a retrospective study, the results of blood cultures and mNGS before antibiotic administration were not able available.

## Data Availability

The raw sequence data reported in this paper have been deposited in the Genome Sequence Archive (Genomics, Proteomics & Bioinformatics 2021) in National Genomics Data Center (Nucleic Acids Res 2022), China National Center for Bioinformation/Beijing Institute of Genomics, Chinese Academy of Sciences (GSA: CRA010141) that are publicly accessible at https://ngdc.cncb.ac.cn/gsa.
